# Spironolactone ameliorates endothelial dysfunction through inhibition of the AGE/RAGE axis in a chronic renal failure rat model

**DOI:** 10.1186/s12882-019-1534-4

**Published:** 2019-09-06

**Authors:** Chun-Cheng Wang, An-Sheng Lee, Shu-Hui Liu, Kuan-Cheng Chang, Ming-Yi Shen, Chiz-Tzung Chang

**Affiliations:** 1 Graduate Institute of Biomedical Sciences, China Medical University, No. 91, Hsueh-Shih Road, Taichung, 40402 Taiwan; 2 Division of Cardiovascular Medicine, Department of Internal Medicine, China Medical University Hospital, No. 2, Yude Road, North District, Taichung, 40447 Taiwan; 3 Cardiovascular Research Laboratory, China Medical University Hospital, Taichung, Taiwan; 4 Department of Medicine, Mackay Medical College, New Taipei, Taiwan; 5 School of Medicine, College of Medicine, China Medical University, No. 91, Hsueh-Shih Road, Taichung, 40402 Taiwan; 6 Department of Medical Research, China Medical University Hospital, Taichung, Taiwan; 7 Department of Nursing, Asia University, Taichung, Taiwan; 8 Division of Nephrology, Department of Internal Medicine, China Medical University Hospital, No 2, Yude Road, North District, Taichung City, 40447 Taiwan

**Keywords:** Advanced glycation end products, Spironolactone, Endothelial dysfunction

## Abstract

**Background:**

Spironolactone can improve endothelial dysfunction in the setting of heart failure and diabetes models. However, its beneficial effect in the cardiovascular system is not clear in the setting of non-diabetic renal failure. We conducted this study to investigate whether spironolactone can ameliorate endothelial dysfunction in a 5/6 nephrectomy model, and to determine the underlying mechanism.

**Methods:**

Twenty-four Sprague-Dawley rats were divided into four groups. A renal failure model was created using the 5/6 nephrectomy method. The four groups included: Sham-operation group (Group1), chronic kidney disease (CKD; Group2), CKD + ALT-711 (advanced glycation end products [AGEs] breaker; Group 3), and CKD + spironolactone group (Group4). Acetylcholine (Ach)-mediated vasodilatation responses were compared between the four groups. To investigate the underlying mechanism, we cultured human aortic endothelial cells (HAECs) for in-vitro assays.

Differences between two groups were determined with the paired student’s t test. Differences between three or more groups were determined through one-way analysis of variance (ANOVA) with post-hoc analysis with LSD method.

**Results:**

Compared with Group 1, Group 2 has a significantly impaired Ach-mediated vasodilatation response. Group 3 and 4 exhibited improved vasoreactivity responses. To determine the underlying mechanism, we performed an in-vitro study using cultured HAECs. We noted significant sirtuin-3 (SIRT3) protein downregulation, reduced phosphorylation of endothelial nitric oxide synthase at serine 1177 (p-eNOS), and increased intracellular oxidative stress in cultured HAECs treated with AGEs (200 μg/mL). These effects were counter-regulated when cultured HAECs were pretreated with spironolactone (10 μM). Furthermore, the increased p-eNOS production by spironolactone was abrogated when the HAECs were pretreated with tenolvin (1 μM), a SIRT3 inhibitor.

**Conclusions:**

Spironolactone could ameliorate endothelial dysfunction in a 5/6 nephrectomy renal failure model through AGEs/Receptor for AGEs (RAGEs) axis inhibition, SIRT3 upregulation, and nicotinamide adenine dinucleotide phosphate oxidase-2 (NOX-2) and its associated intracellular oxidative stress attenuation.

**Electronic supplementary material:**

The online version of this article (10.1186/s12882-019-1534-4) contains supplementary material, which is available to authorized users.

## Background

Chronic kidney disease (CKD) is a well-known risk factor for cardiovascular disease. Approximately half of the uremia patients die of cardiovascular disease. [1] The association between CKD and cardiovascular disease cannot be explained by conventional cardiovascular disease risk factors. Accelerated atherosclerosis has been observed in the setting of CKD and has been proposed as a reason for higher-than expected death rate in patients with CKD. [2] The explanation for the association between CKD and cardiovascular disease may be attributed to increased oxidative stress. [3, 4] Advanced glycation end products (AGEs) are formed by a non-enzymatic reaction between amino acids and sugar called the Maillard reaction. [5] The formation of AGEs requires the presence of increased oxidative stress. [6] Clinically, increased AGEs can be observed in the setting of aging, smoking, diabetes, and CKD. Studies have implied that tissue accumulation of AGEs could result in arterial stiffness, endothelial dysfunction and atherosclerosis. [7–10] Hence, AGEs may play a major role in CKD-related cardiovascular disease.

Spironolactone is a mineralocorticoid receptor antagonist. Previous study proposed that spironolactone could ameliorate endothelial dysfunction in a heart failure rat model. [11] However, the question whether spironolactone can have overall long-term beneficial outcomes in patients with CKD remains controversial. The potential beneficial cardiovascular effect should weigh against the risk of hyperkalemia in the setting of renal failure. [12] Recently, a clinical trial indicated that low-dose spironolactone (25 mg daily) could provide better cardiovascular outcomes in uremia patients and the plasma potassium levels were not significantly increased. [13] However, the potential mechanism through which spironolactone engenders cardiovascular benefits in patients with CKD remained elusive. To investigate this issue, we conducted an animal study and used the Sprague-Dawley rat, a model which has been applied in all aspects of biomedical research. We used 5/6 radical nephrectomy as an experimental renal failure model.

We hypothesized that treatment with spironolactone in a 5/6 nephrectomy renal failure rat model could ameliorate endothelial dysfunction, and that the beneficial effect of spironolactone may involving inhibition of the AGEs/Receptor for AGEs (RAGE) axis and attenuation of cellular oxidative stress.

## Methods

### Animals and experimental design

Twenty-four male Sprague-Dawley rats, weighing 180–200 g, from BioLASCO Taiwan Co., Ltd. (Taipei, Taiwan) were housed at the animal facility of China Medical University under a 12-h light-dark cycle and had free access to a standard rat chow diet and tap water. The food and water intake, their activity, and urine output were observed daily throughout the experiment. After an acclimization period of 2 weeks, the animals were randomly divided into four groups.

Group 1 (Control group, *n* = 6) comprised rats that received a sham-operation.

Group 2 (CKD group, n = 6) contained rats that were anesthetized with pentobarbital sodium (35 mg/kg, administered intraperitoneally) and received a two-stage 5/6 nephrectomy operation. (The upper and the lower poles of the left kidney, as well as the whole right kidney were removed) [14, 15] The interval between the two index operation was 2 weeks. After the procedure, the rats were given a standard rat chow diet and tap water ad libitum for 2 months. After the two-month post-operation period, blood samples were collected to measure plasma blood urea nitrogen (BUN), creatinine, and AGEs levels. The rats were then treated with same volume of vehicle by oral gavage for the subsequent one month.

Group 3 (CKD + AGEs breaker [ALT-711], *n* = 6) comprised rats that received the same two-stage 5/6 nephrectomy operation. The rats were given a standard rat chow diet and tap water ad libitum for 2 months post-operation. Blood samples were collected. After the 2-month post-operation period, the rats were treated with ALT-711 (3 mg/kg/day) by oral gavage for 1 month.

**Fig. 1:**
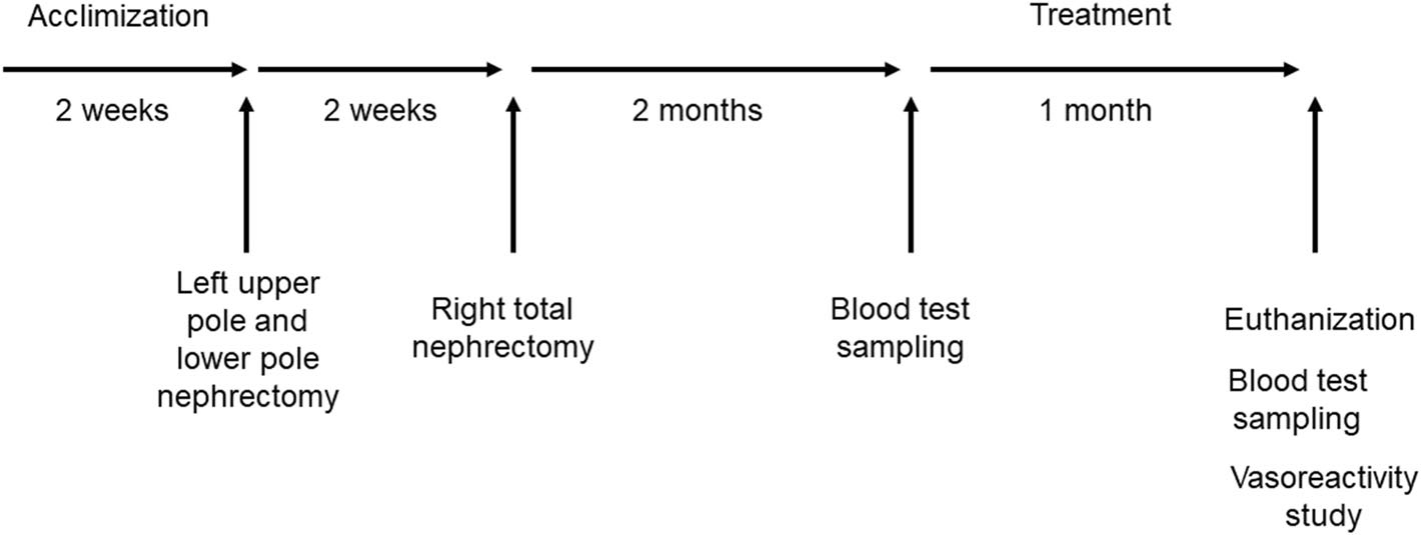
Animal study protocol. Twenty-four Sprague-Dawley rats were divided into four groups [Group 1: Sham-operation group, Group 2: Chronic kidney disease (CKD) group, Group 3: CKD + ALT-711 (3 mg/kg/day) group, Group 4: CKD + spironolactone (7 mg/kg/day)]. During the treatment period, groups 1 and 2 were treated with the same volume of vehicle by oral gavage; group 3 and group 4 were treated with advanced glycation end products (AGEs) breaker (ALT-711) 3 mg/kg/day, and spironolactone 7 mg/kg/day by oral gavage, respectively

Finally, Group 4 (CKD + spironolactone, n = 6) contained rats that received the same two-stage 5/6 nephrectomy operation. The rats were given a standard rat chow diet and tap water ad libitum for 2 months post-operation. Blood samples were collected. After the 2-month post-operation period, the rats were treated with spironolactone (7 mg/kg/day) by oral gavage for 1 month (Fig. 1).

At the end of the experiment, body weights were measured in all animals, and blood pressure levels were measured in all conscious animals by using the tail-cuff method. The rats were euthanized with CO2 inhalation. The remnant kidneys and hearts were removed and weighed. The thoracic aorta was immediately removed for vascular reactivity study, and a portion of the thoracic aorta was fixed in 10% neutral buffered formalin for immunohistochemical analysis.

All animal studies adhere to the National Institutes of Health Guide for the Care and Use of Laboratory Animals, Eighth Edition. All animal study protocols were approved by the Institutional Animal Use and Care Committee of China Medical University (protocol number: 104–272).

### Vascular reactivity study

#### Vessel preparation

The thoracic aorta was rapidly removed and excised into 3 mm segments. A sample was transferred onto an arteriograph system (Radnoti M1000; Covina, California, USA) for measuring vascular tension force. To mount the vessel segments, two individual parallel wires were passed through the vessel lumen, and attached to the support brackets. One bracket was connected to an isometric force transducer. The vessel ring was bathed in a 5-mL chamber containing Kreb’s ringer solution at 37 °C. Determining arterial reactivity.

To evaluate endothelium-dependent arterial relaxation, aortic rings were precontracted with phenylephrine at a concentration of 10^− 6^ mol/L. As the arterial contraction reached a plateau and stabilized, acetylcholine (Ach) was added cumulatively at concentrations in the range of 10^− 8^ - 10^− 5^ mol/L. To evaluate endothelium-independent arterial relaxation, the endothelial layer of the aortic ring was denuded, and precontracted with phenylephrine at a concentration of 10^− 6^ mol/L. Sodium nitroprusside (SNP) was then added cumulatively at concentrations in the range of 10^− 9^ - 10^− 6^ mol/L.

#### Immunohistochemical staining

Sections of the thoracic aorta were fixed in 10% neutral buffered formalin for 24 h. After fixation, a tissue block was embedded in paraffin and sectioned with a microtome. Subsequently, the tissue sections were mounted on glass slides and dried to remove any water. Before being stained with primary and secondary antibodies, the tissue sections on the glass slides were deparaffinized with xylene, ethanol before rehydration. To remove the formation of methylene bridges during fixation, heat- induced epitope retrieval was applied. The tissue sections were stained with a primary antibody such as mouse anti-endothelial nitric oxide synthase (eNOS) antibody (1:50; Abcam, Cambridge, MA, USA) or mouse anti–phospho-eNOS antibody (p-eNOS) (1:50; Abcam, Cambridge, MA, USA), or mouse anti-RAGEs antibody (1:50; Abcam, Cambridge, MA, USA) at 4 °C overnight. Subsequently, the stained sections were washed with phosphate-buffered saline (PBS) twice and incubated with a horseradish peroxidase (HRP)-conjugated mouse IgG secondary antibody (1500; GeneTex, California, USA) at room temperature for 1 h. Images were acquired using a Nikon E600 light microscope (Nikon, Tokyo, Japan; magnification, 200X).

### Cell culture

Human aortic endothelial cells (HAECs) were purchased from PromoCell (Heidelberg, Germany). The HAECs were cultured in the endothelial cell growth medium MV2 (PromoCell GmbH, Heidelberg, Germany) supplemented with 10% heat-inactivated fetal bovine serum and 1% penicillin-streptomycin(Thermo Fisher Scientific, MA, USA) at 37 °C in a 5% CO2 atmosphere. The medium was replaced every 2 days. Passage 5–6 was used for this study.

#### 3-(4,5-Dimethylthiazol-2-yl)-2,5-diphenyltetrazolium bromide (MTT) assay

The HAECs were plated on a 96-well plate at a density of 5000–10,000/well. The cells were treated with the vehicle, AGEs (Sigma Aldrich, Merck KGaA, Darmstadt, Germany) or bovine-serum albumin (BSA) (Sigma-Aldrich, Merck KGaA, Darmstadt, Germany) at concentrations of 100, 200 and 500 μg/ml and were incubated for 24 h. After the incubation period, the HAECs were treated with 20 μL of 5 mg/mL MTT reagent and incubated for 4–6 h at 37 °C. The media were carefully removed subsequently. Dimethyl sulfoxide (DMSO)(Sigma Aldrich, Merck KGaA, Darmstadt, Germany) 100 μL was added to each well and followed by thorough mixing to dissolve formazan crystals. Subsequently, the cells were agitated on a plate shaker for 5 min. Absorbance was measured at 570 nm using a TECAN Infinite M1000 plate reader (Thermo Fisher Scientific, MA, USA). Results were compared with the control group.

### Measurements of cytosolic and mitochondrial oxidative stress

The HAECs were seeded in a 96-well plate at a density of 1.4 × 10^4^ cells per well. The cells were pretreated with AGEs neutralizing antibody 50 μg/mL, N-acetylcysteine 20 mM, and spironolactone (Sigma-Aldrich, Merck KGaA, Darmstadt, Germany) at concentrations of 0.1 μM, 1 μM, and 10 μM for 30 min. The cells were then treated with AGEs 200 μg/mL and incubated for 24 h. For the control group, the cells were treated with the vehicle. In each well, the cells were rinsed three times with PBS (Thermo-Fisher Scientific, MA, USA) for 5 min. Subsequently, 100 μL of 2.5 μM 2′,7′-Dichlorodihydrofluorescein diacetate (DCFH-DA) (Millipore, Merck KGaA, Darmstadt, Germany) or 100 μL of 5 μM MitoSox red (Invitrogen, CA, USA) was added to each well, according to the study design, after which the cells were incubated for 30 min in the dark. Cytosolic and mitochondrial oxidative stress levels were next quantified by measuring the fluorescence density at excitation and emission wavelengths of 485 nm and 530 nm, respectively, using the TECAN Infinite M1000 plate reader (Thermo Fisher Scientific, MA, USA).

### Western immunoblotting

Protein concentrations were determined using a BCA protein assay kit. Equal amounts (20 μg/lane) of protein were separated using 10% sodium dodecyl sulphate-polyacrylamide gel electrophoresis (SDS-PAGE) at 70–120 V. The proteins were next transferred to polyvinylidene difluoride (PVDF) membranes through electroblotting for 60 min at 100 V. The transferred membranes were then blocked with 5% non-fat milk [1 g non-fat milk dissolved in 20 mL Tris-buffered saline with 0.05% Tween-20 (TBST)] for 2 h on a shaking table. Subsequently, the membranes were incubated with primary antibodies, including anti-eNOS (1:1000; Abcam, MA, USA), p-eNOS Ser 1177 (1:1000; Abcam, MA, USA), RAGEs (1:1000; Abcam, MA, USA), sirtuin-3 (SIRT3; 1:1000; SantaCruz, Texas, USA), and glyceraldehyde 3-phosphate dehydrogenase (GAPDH; 1:2000; GeneTex, CA, USA) at 4 °C overnight on a shaking table. The membranes were then probed with their respective secondary antibodies conjugated with horseradish peroxidase including rabbit IgG antibody and mouse IgG antibody (1:10000; GeneTex, CA, USA) at room temperature for 50 min. After the probing process, each of the membranes was washed three times with TBST for 5 min. The bands were then visualized using an enhanced chemiluminescence detection kit (ECL kit) (Millipore, Merck KGaA, Darmstadt, Germany). The protein bands were quantified using Image J software.

### Confocal microscopy

Intact cells were seeded on a 8-well chamber slide at a density of 1.7 × 10^4^ cells/well and cultured overnight. On the second day, different groups of the cultured cells were treated with the vehicle, AGEs 200 μg/mL, AGEs 200 μg/mL + spironolactone 10 μM, and AGEs 200 μg/mL + spironolactone 10 μM + Tenovin-6 1 μM; the treated cells were incubated for 24 h. Next, the cells were fixed with 4% paraformaldehyde and incubated with the diluted primary antibody rabbit anti- nicotinamide adenine dinucleotide phosphate oxidase (NOX-2; 1:100; Thermo-Fisher Scientific, MA, USA), followed by incubation overight at 4 °C. Nuclei were stained with 4′, 6′-diamidino-2-phenylindole (DAPI) (excitation wavelength, 543 nm; emission wavelength, 573 nm). Subsequently, the cells were probed with a fluorochrome-conjugated secondary antibody (Donkey anti-Rabbit IgG, Alexa Fluor 594;1:500; Thermo-Fisher Scientific, MA, USA) and were incubated for 1 h at room temperature. Cells on different chamber slides were examined in a confocal imaging system (Zeiss LSM 510 META, Heidelberg, Germany).

To avoid subjective bias when assessing the results of the animal experiments and cell culture assays, the analyses of the study results were performed by CKC, SMY, and CCT, who were blinded to the experimental conduction.

### Statistical analysis

Continuous variables are expressed as mean ± standard error from at least three experiments. Differences between before and after treatments in the same group were determined with the paired student’s t test. Differences between three or more groups were determined through one-way analysis of variance (ANOVA) with post-hoc analysis with LSD method. A *p* value < 0.05 was considered as statistically significant. All statistical analyses were performed using SPSS 16.0, IBM, Chicago IL, USA.

## Results

**Fig. 2:**
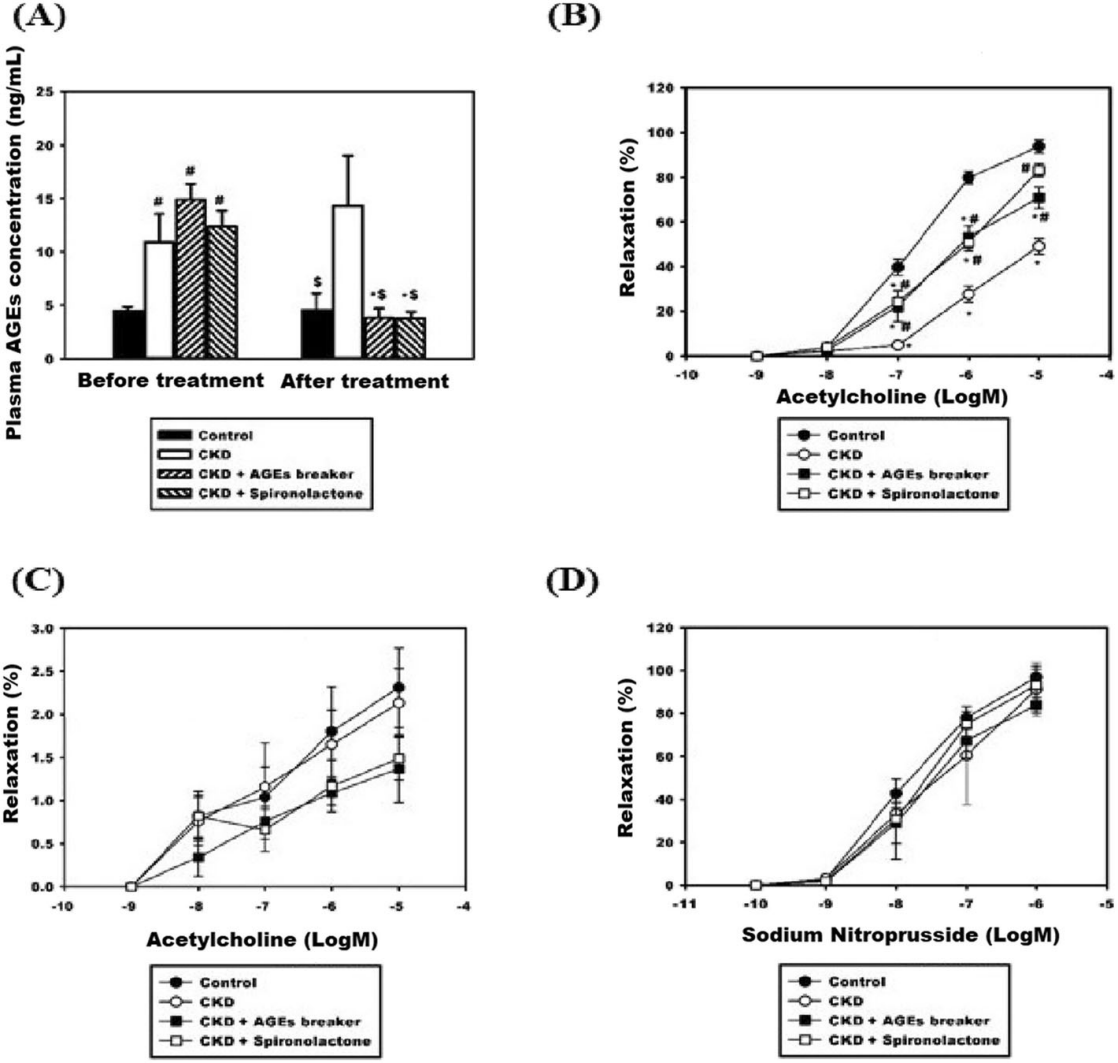
(**a**) Comparisons of plasma advanced glycation end products (AGEs) levels between groups and treatments. ^*^ Before versus. After treatments, *p* < 0.05; ^#^ any groups compared with the control group before treatment, *p* < 0.05; ^$^ any groups compared with the control group after treatment, *p* < 0.05; (**b**) Comparisons of differences of Acetylcholine (Ach)-related vasorelaxation between groups. ^*^ any groups compared with the control group (Group 1), *p* < 0.05; ^#^ any groups compared with the CKD group (Group 2), *p* < 0.05. (**c**) Comparisons of inhibitory effect of N(omega)-Monomethyl-L-Arginine Acetate (L-NMMA) on the Ach-related vasorelaxation between groups. (**d**) Comparisons of sodium nitroprusside (SNP)-related vasorelaxation between groups

**Fig. 3:**
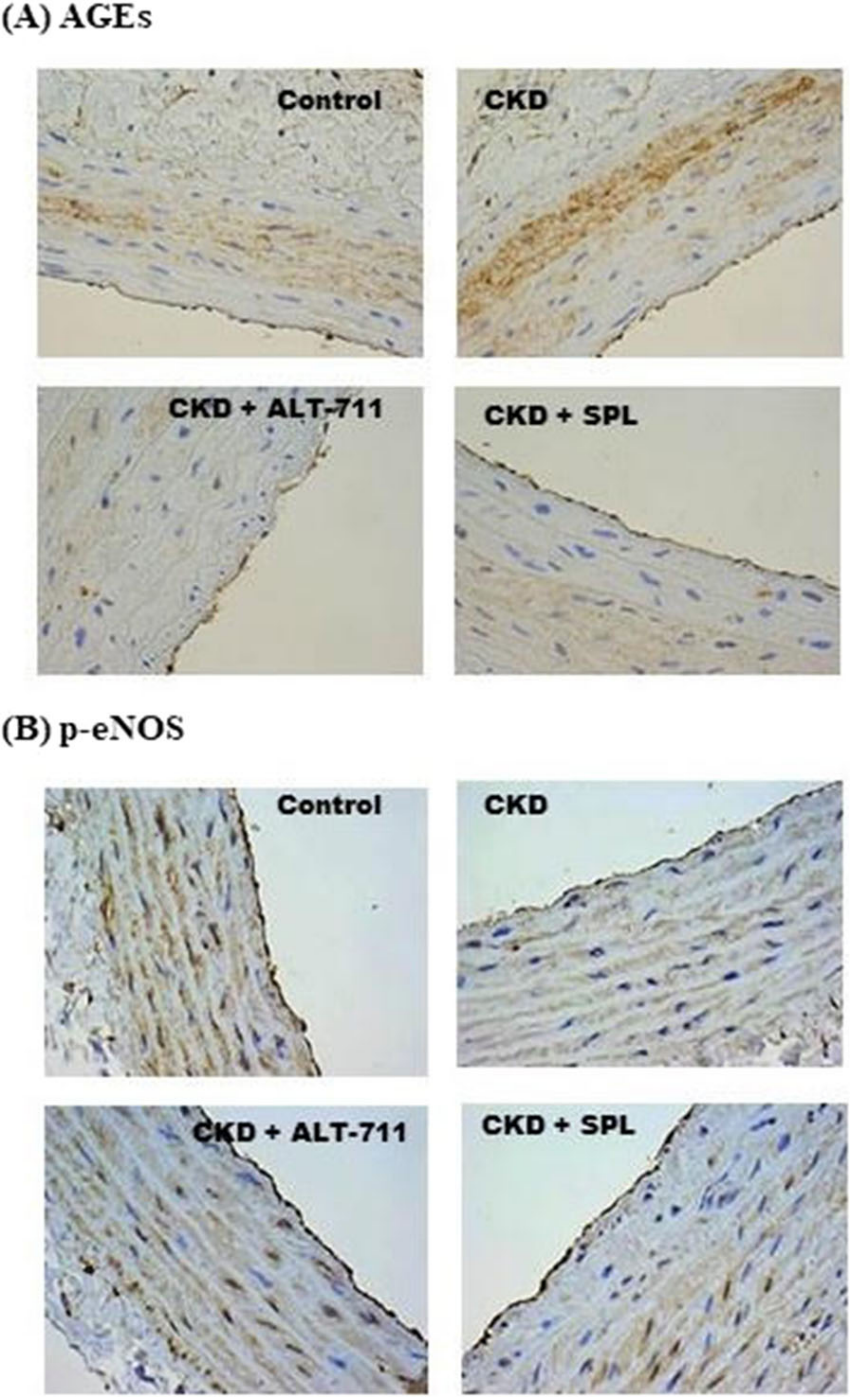
Results derived from the immunohistochemical stain of thoracic aortas obtained from the Sprague-Dawley rats. (**a**) Accumulation of the advanced glycation end products (AGEs) in thoracic aorta tissue was higher in the chronic kidney disease (CKD) group compared with it was in the control, CKD + ALT-711, and CKD + spironolactone groups. (**b**) Amount of phospho-endothelial nitric oxide synthase (p-eNOS) in the thoracic aorta tissue was lower in the CKD group compared with it was in the control, CKD + ALT-711, and CKD + Spironolactone groups

**Fig. 4:**
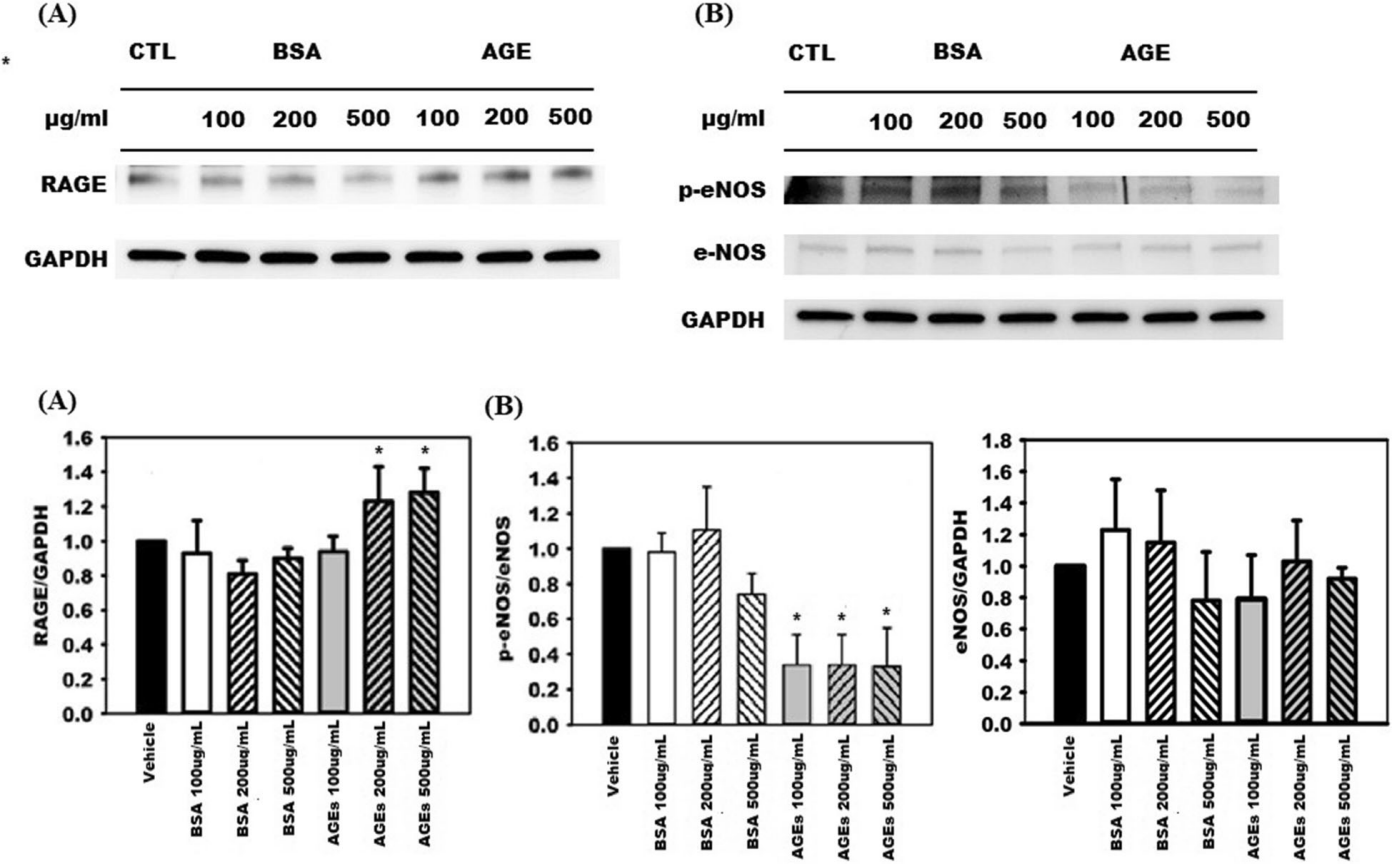
Advanced glycation end products (AGEs) stimulated (**a**) expression of receptors for advanced glycation end products (RAGEs) and inactivated (**b**) phosphorylation of endothelial nitric oxide synthase (eNOS) in a dose-dependent manner; * any groups compare with the vehicle group, *p* < 0.05

**Fig. 5:**
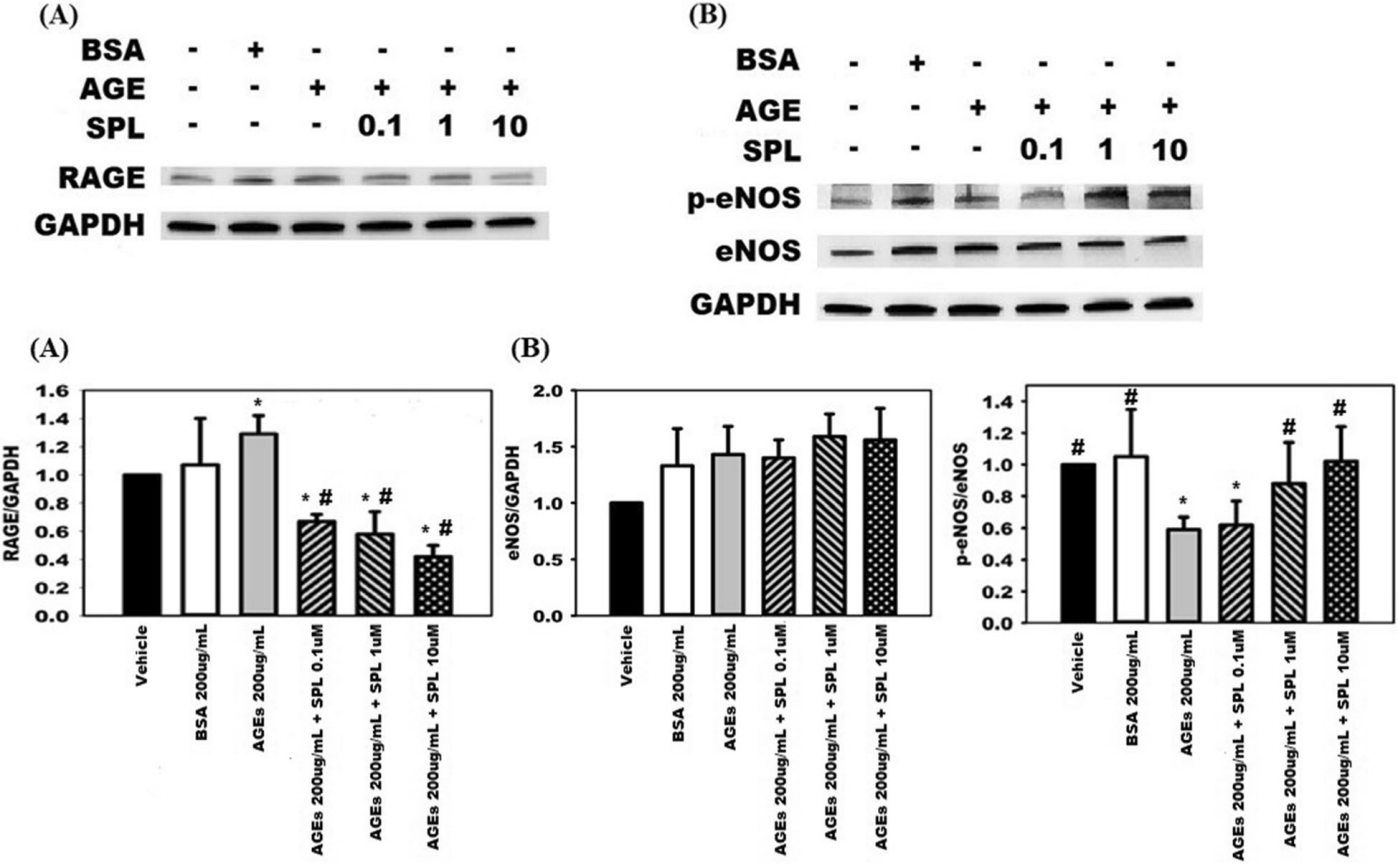
Spironolactone (**a**) suppresses the receptor for advanced glycation end products (RAGEs) expression induced by advanced glycation end products (AGEs) stimulation and (**b**) increases the phosphorylation of endothelial nitric oxide synthase (p-eNOS) in a dose-dependent manner. * any groups compared with the vehicle group, *p* < 0.05; # any groups compared with the AGEs group, *p* < 0.05

**Fig. 6:**
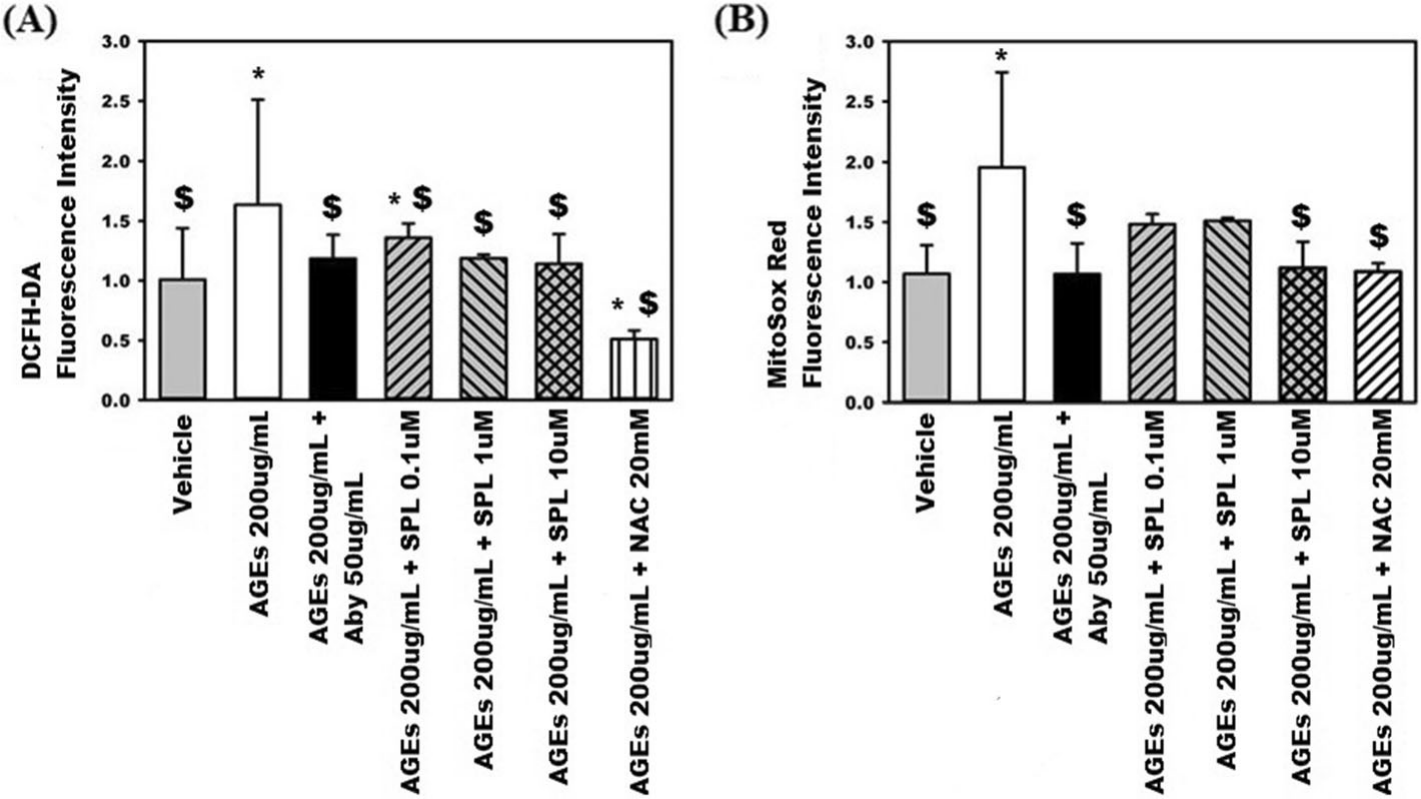
Spironolactone (SPL) reduces (**a**) cytosolic and (**b**) mitochondrial reactive oxidative species (ROS) production in human aortic endothelial cells (HAECs) stimulated by advanced glycation end products (AGEs) at a concentration of 200 μg/ml. Pre-treatment of AGEs-stimulated HAECs with anti-AGEs neutralizing antibody (Aby) at a concentration of 50 μg/ml or N-acetylcysteine (NAC) at a concentration of 20 mM was used as a positive control. * any groups compared with the vehicle, *p* < 0.05; $ any groups compared with the AGEs (200 μg/mL)

**Fig. 7:**
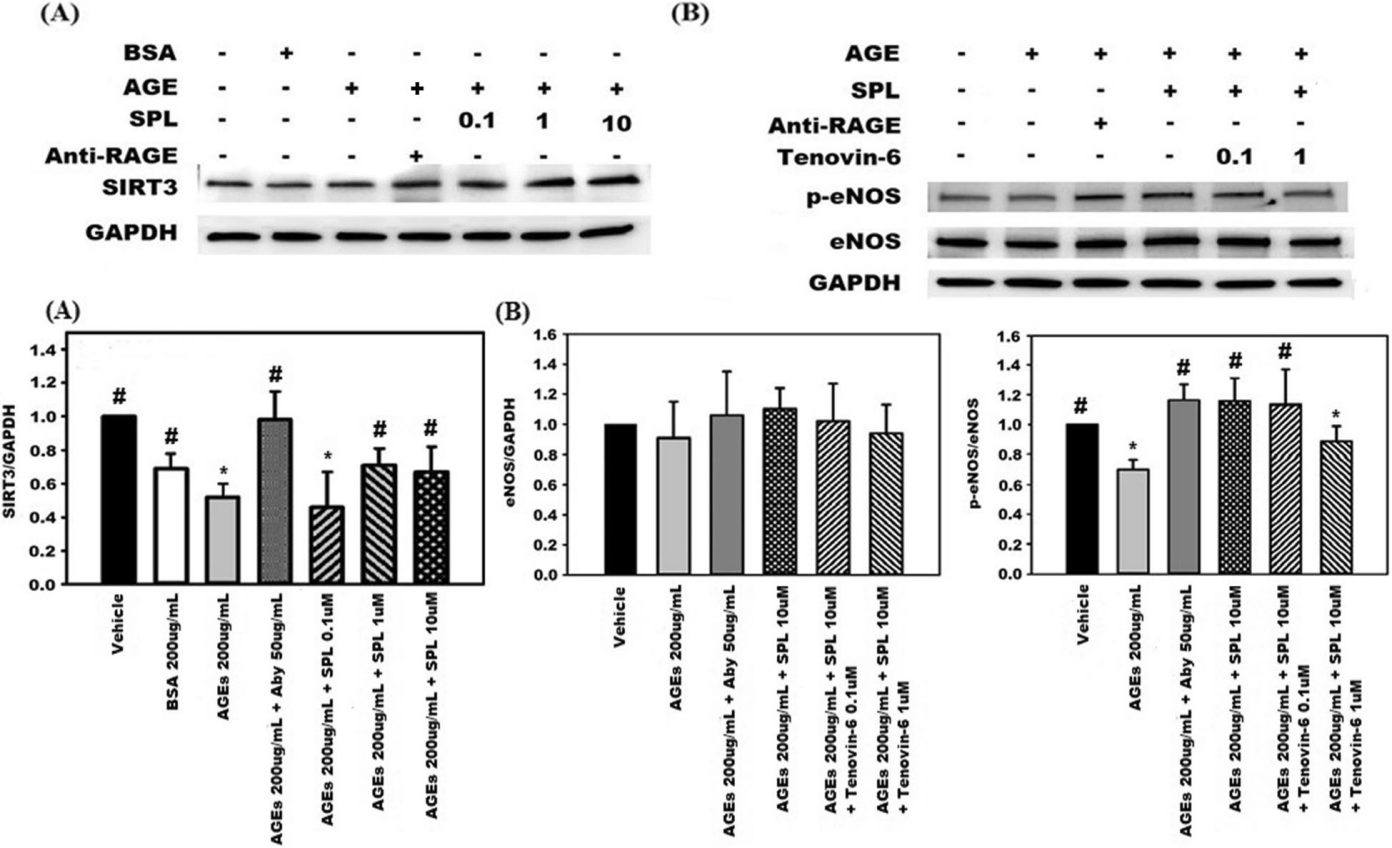
Spironolactone (SPL) improves endothelial dysfunction in advanced glycation end products (AGEs) –stimulated human aortic endothelial cells (HAECs) through sirtuin-3 (SIRT3) protein mediation. (**a**) Downregulation of SIRT3 was noted in AGEs-stimulated HAECs. The addition of SPL at concentrations of 1 μM and 10 μM improved the SIRT3 protein expression. (**b**) SPL increased phospho-endothelial nitric oxide synthase (p-eNOS) activation at a concentration of 10 μM in AGEs-stimulated HAECs. However, pretreatment of AGEs-stimulated HAECs with tenovin-6 at a concentration of 1 μM abrogated the effect of SPL on the p-eNOS activation. * any groups compared with the vehicle, *p* < 0.05; # any groups compared with the AGEs group, *p* < 0.05

**Fig. 8:**
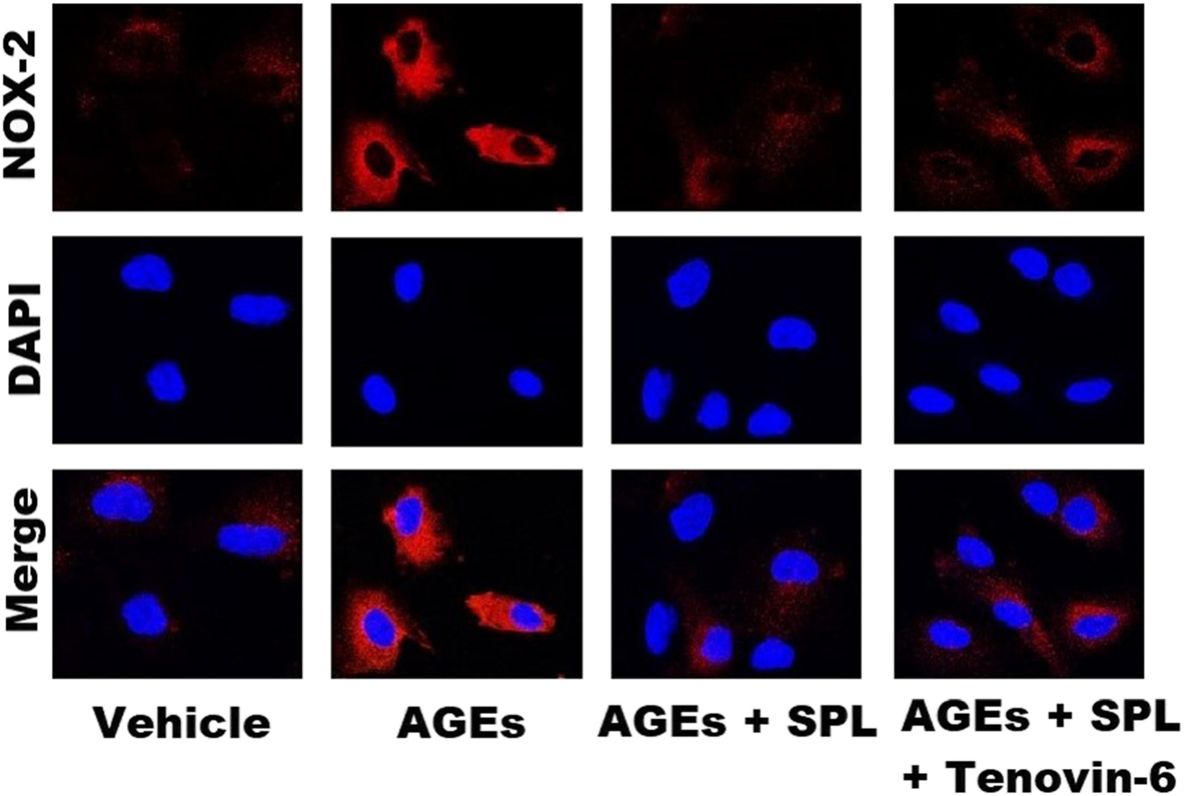
Application of confocal microscopy to illustrate differences in fluorescence intensity of nicotinamide adenine dinucleotide phosphate oxidase (NOX-2) between different groups. Human aortic endothelial cells (HAECs) treated with the vehicle were used as a negative control group. Compared with the AGEs-stimulated group, pre-treatment with spironolactone (10 μM) in AGEs-stimulated HAECs resulted in a lower fluorescence intensity of NOX-2. Pre-treatment with tenovin-6 (1 μM; the AGEs + spironolactone + tenovin-6 group) resulted in a higher NOX-2 fluorescence intensity than did the AGEs + Spironolactone group

Table 1 presents comparisons of the four groups in terms of body weights, blood pressure, and renal functions before and after treatments. No significant differences of body weights were noted between the four groups. Compared with Group 1, both of the systolic blood pressure and diastolic blood pressure measurements were significantly higher in the Group 2–4. However, no significant differences of systolic blood pressure and diastolic blood pressure measurements were noted between Group 2–4. Compared with Group 1, the other three groups had significantly higher plasma BUN levels and plasma creatinine levels. In Groups 3 and 4, treatments with the AGEs breaker (ALT-711) or spironolactone failed to improve renal function in the 5/6 nephrectomy rat model (Group 3 creatinine: 0.90 ± 0.22 mg/dL vs. 1.82 ± 0.76 mg/dL, *p* = 0.06; Group 4 creatinine: 1.08 ± 0.33 mg/dL vs. 1.32 ± 0.33 mg/dL, *p* = 0.19). In addition, no significant differences of plasma creatinine levels after the treatments between Groups 2–4 were noted. Plasma AGEs levels measured before and after the treatments during the study period were compared, as illustrated in Fig. 2a. Before the treatments, the plasma AGEs levels of Groups 2–4 were significantly higher than that of Group 1. (Group 2: 11.39 ± 2.43 ng/mL; Group 3: 12.25 ± 3.03 ng/mL; Group 4: 11.24 ± 1.67 ng/mL vs. Group 1: 4.32 ± 0.71 ng/mL; *p* < 0.01). After the treatment, the plasma AGEs level of Group 2 was significantly higher than the other three groups. (Group 1: 4.71 ± 1.07 ng/mL; Group 3: 4.01 ± 1.05 ng/mL; Group 4: 4.40 ± 1.03 ng/mL vs. Group 2: 14.04 ± 3.89 ng/mL; *p* < 0.01). Furthermore, the plasma AGEs levels before and after the treatments were compared in the four groups. Specifically, the plasma AGEs levels observed after the treatments were significantly lower than those observed before the treatments plasma AGEs levels in Group 3 (4.01 ± 1.05 vs. 12.25 ± 3.03 ng/mL; *p* < 0.01) and in Group 4 (4.40 ± 1.03 vs. 11.24 ± 1.67 ng/mL; *p* < 0.01). In Group 2, we observed no significant difference in plasma AGEs levels before and after the treatment (14.04 ± 3.89 vs. 11.39 ± 2.43 ng/mL, *p* = nonsignificant). We also explored the differences in flow-mediated vasodilatation responses between the four groups, as illustrated in Fig. 2b. In all four groups, we noted progressively increased vasorelaxation in response to Ach stimulation in a dose-dependent manner (10^− 8^-10^− 5^ M). However, the percentages of Ach-stimulated vasorelaxation in Group 2 were significantly lower than those in the other three groups. To further investigate the contribution of endothelial layers to the Ach-stimulated vasorelaxation observed in the four groups, the endothelial layers were pretreated with N(omega)-monomethyl-L-arginine acetate (L-NMMA, 10^− 4^ M). The Ach-stimulated vasorelaxation responses were comparable and significantly impaired in all four groups. (Fig. 2c) To evaluate endothelium-independent vasodilatation, segments of the thoracic aorta obtained from the four groups were denuded and treated with SNP. The extent of vasodilatation in response to SNP stimulation increased in a dose-dependent manner (10^− 9^-10^− 6^ M) and this observation was prevalent across all four groups. No significant differences of vasodilatation could be noted between the four groups.(Fig. 2d) In summary, treating 5/6 nephrectomy rats with spironolactone could improve endothelium-dependent vasodilatation. The thoracic aorta segments obtained from the four groups were subjected to immunohistochemical stain and the stain results are shown in Fig. 3 and Additional file 1. Compared with the other three groups, Group 2 had higher levels of tissue accumulation of AGEs (Fig. 3a) and RAGE (Additional file 1a). Group 2 had a lower level of tissue accumulation of p-eNOS than did the other three groups (Fig. 3b). The levels of tissue accumulation of eNOS were comparable across all four groups. (Additional file 1b) To further elucidate whether spironolactone could inhibit the AGEs/RAGE axis, we pre-treated the AGEs-stimulated HAECs with spironolactone and evaluated its effects on eNOS, RAGE, and oxidative stress levels. The MTT assay was applied to determine the viability of the HAECs in response to treatments with different concentration gradients of AGEs, BSAs (0, 100, 200, and 500 μg/mL) or spironolactone (0, 0.1, 1 and 10 μM) (Additional file 2). Compared with the control group (0 μg/mL), no significant differences in cell viability were noted when the HAECs were treated with AGEs, BSAs at a concentration of 200 μg/dL or spironolactone at a concentration of 10 μM for 24 h. To investigate the effect of AGEs stimulation, the HAECs were treated with AGEs at different concentrations (100, 200, and 500 μg/mL) for 24 h, and the results were compared with those observed for BSA, and the vehicle (Fig. 4). Compared with BSA and the vehicle, treatment with AGEs significantly increased expression of RAGE and reduced phosphorylation of eNOS. The expression of eNOS was comparable between the groups. This suggests that stimulation of HAECs by AGEs can provide a positive feedback loop and augment an inflammatory cascade initiated by the AGEs/RAGE axis. To evaluate whether spironolactone has an inhibitory effect on the AGEs/RAGE axis, we pre-treated the AGEs-stimulated HAECs with spironolactone at different concentrations (0.1, 1, and 10 μM) and the results are presented in Fig. 5. Compared with the AGEs-stimulated HAECs, pre-treatment with spironolactone resulted in a progressively decreased expression of RAGE and increased phosphorylation of eNOS in a concentration-dependent manner. This suggests that spironolactone may ameliorate endothelial dysfunction through inhibition of the AGEs/RAGE axis. Previous studies have suggested that increased oxidative stress plays a major role in endothelial dysfunction. We treated the HAECs with the fluorescent probe DAFH-DA (2.5 μM) and MitoSox red (5 μM) and subsequently measured cytosolic hydrogen peroxide and mitochondrial superoxide in each group by using a microplate reader. (Fig. 6) Compared with the vehicle group, the AGEs group exhibited significantly increased levels of cytosolic and mitochondrial oxidative stress. However, both the cytosolic and mitochondrial oxidative stress reduced progressively when the AGEs-stimulated HAECs were pre-treated with spironolactone in a dose-dependent manner. This implies that spironolactone may attenuate intracellular oxidative stress induced by the AGEs/RAGE axis. To further provide insights into how the AGEs/RAGE axis regulates cellular oxidative stress, we evaluated the association between the AGEs/RAGE axis, SIRT3, and NOX (Figs. 7 and 8). Compared with the AGEs-treated group, the expression of SIRT3 was significantly increased when the AGEs-stimulated HAECs were pre-treated with spironolactone (1, and 10 μM) as shown in Fig. 7a. The effect of SIRT3 on endothelial dysfunction is presented in Fig. 7b. Compared with the AGEs-stimulated HAECs, the phosphorylation of eNOS increased significantly when the AGEs-stimulated HAECs were pre-treated with spironolactone (10 μM). However, when the HAECs were pre-treated with the SIRT3 inhibitor, tenovin-6 (1 μM), the effect of spironolactone on the phosphorylation of eNOS was abrogated. This implies that spironolactone ameliorated the AGEs/RAGE associated endothelial dysfunction through the upregulation of SIRT3. NOX-2 (a prototype of NOX) is a major source of cytosolic oxidative stress and plays a major role in atherosclerosis. We therefore investigated the interaction between SIRT3 and NOX-2. (Fig. 8) Confocal microscopy revealed that the fluorescence intensity of NOX-2 was reduced in the AGEs + spironolactone treated group compared with the group treated with AGEs. However, when the HAECs were pre-treated with tenovin-6, the fluorescence intensity of NOX-2 was increased compared with that observed in the group treated with AGEs + spironolactone. This implies that suppression of SIRT3 may upregulate the NOX-2 protein. Spironolactone may downregulate the NOX-2 through SIRT3 upregulation.

**Table 1:** Comparisons of different groups in terms of renal function before and after treatments

	BUN before treatment (mg/dL)	BUN after treatment (mg/dL)	Cr before treatment (mg/dL)	Cr after treatment (mg/dL)	Body weight (g)	SBP (mmHg)	DBP (mmHg)
Control	15.83 ± 1.6	15.33 ± 3.88	0.73 ± 0.05	0.7 ± 0.18	588.83 ± 44.74	125.50 ± 5.58	73.33 ± 4.18
CKD	35.15 ± 4.18^#^	45.83 ± 4.96*^,$^	0.82 ± 0.20	1.7 ± 0.43*^,$^	551.67 ± 25.10	164.83 ± 7.31^$^	86.33 ± 4.5^$^
CKD + ALT-711	32.5 ± 7.48^#^	46 ± 17.32*^,$^	0.90 ± 0.22	1.82 ± 0.76^$^	585.67 ± 48.30	161.83 ± 5.95^$^	83.50 ± 4.72^$^
CKD + SPL	37.5 ± 8.17^#^	43.83 ± 18.09^$^	1.08 ± 0.33	1.32 ± 0.33^$^	562.50 ± 54.24	165.17 ± 6.18^$^	85.00 ± 3.58^$^

*BUN* blood urea nitrogen, *Cr* creatinine, *CKD* chronic kidney disease, *SPL* spironolactone, *SBP* systolic blood pressure, *DBP* diastolic blood pressure, ^*^ Before treatment versus. After treatment, *p* < 0.05; ^#^ any groups compared with the control group before treatment, *p* < 0.05, ^$^ any groups compared with the control group after treatment, *p* < 0.05

## Discussion

The major findings of our study include the following: First, spironolactone ameliorated endothelial dysfunction in a 5/6 nephrectomy renal failure model. Second, spironolactone could inhibit the AGEs/RAGE axis through the downregulation of RAGE protein expression, thereby disrupting the self-promoting positive feedback loop of the AGEs/RAGE axis and its associated inflammatory cascade. [16, 17] Third, spironolactone may reverse the downregulatory effect of SIRT3 caused by the AGEs/RAGE axis, thereby reducing mitochondrial reactive oxygen species (ROS) production, and the NOX-2 expression. The overall oxidative stress reduction may result in eNOS activation.

### Spironolactone improves endothelial dysfunction in the setting of CKD

Previous studies have suggested that mineralocorticoid receptor antagonist could ameliorate endothelial dysfunction in the setting of heart failure, diet-induced obesity, and a streptozocin-induced diabetic model. [11, 18, 19] However, whether spironolactone can ameliorate endothelial dysfunction in the setting of non-diabetic nephropathy is unclear. Our study provides evidence that spironolactone could ameliorate endothelial dysfunction, an early indicator of atherosclerosis, in a non-diabetic renal failure model. Unlike previous studies [20, 21], our study failed to demonstrate that treatment with spironolactone could improve renal function. Our study design may explain this discrepancy, we used 5/6 radical surgical nephrectomy to create the renal failure model, and such an advanced stage of renal failure renders treatment with spironolactone ineffective. Though our study failed to demonstrate significant differences of plasma creatinine levels after the treatment between Groups 2,3 and 4. A lower renal function decline rate could be observed in Group 4 in comparison with those of Group 2 and 3. This indicates that spironolactone and ALT-711 may have differential effects and mechanism on renal function. Our study cannot exclude the possibility that by application of other renal failure models, spironolactone and ALT-711 may improve endothelial dysfunction through improving renal function by different pathways. However, based on current evidence, our study suggests that instead of improving renal function, spironolactone ameliorated endothelial dysfunction in the 5/6 nephrectomy model through directly antagonizing the vasculopathy effect caused by uremic toxin. AGEs are well-known uremic toxins, and our study suggest that spironolactone may alleviate the AGEs-induced vasculopathy.

### Mitochondrial protection role of SIRT3

The sirtuins belong to a highly conserved family of NAD^+^- dependent enzymes that regulate lifespan in lower organisms. The role of sirtuins to prevent senescence, insulin resistance, and vascular atherosclerosis has been proposed.^20^ The SIRT3 is present in mitochondria and has been proposed to reduce mitochondrial ROS production and maintain mitochondrial integrity. SIRT3 can deacetylate global mitochondrial proteins, such as acetyl coenzyme A synthetase 2, and complex 1 of the electron transport chain, and activate manganese superoxide dismutase, thereby reducing mitochondrial ROS production. [22–25] Furthermore, SIRT3 can deacetylate FOXO3, thereby protecting mitochondria from oxidative damage. [26] Our finding that the AGEs/RAGE axis activation could downregulate the production of SIRT3 in HAECs is in line with those in the literature. [27] Our study further suggests that pretreatment with spironolactone in the HAECs can reverse SIRT3 production and reduce mitochondrial oxidative stress.

### Interaction between NOX and mitochondrial ROS

As illustrated in Fig. 8, NOX2 expression increased when HAECs were pretreated with tenovin-6 (SIRT3 inhibitor). This may be explained by the connection between mitochondrial ROS production and NOX. [28] A crosstalk between NOX and mitochondrial ROS production has been a well-known example of ROS-induced ROS release (RIRR). ROS generated from mitochondria could stimulate phosphoinositide 3-kinase and translocate the Rac-1, thereby activating NOX. The ROS generated from mitochondria may be transient and their stimulation of NOX may provide a more sustained, self-amplified ROS production. [28] Our findings indicate that AGEs/RAGE axis activation could downregulate the SIRT3 expression and increase mitochondrial ROS production. Such increased mitochondrial ROS production may further stimulate NOX2 production, further augment cytosolic ROS production, and ultimately lead to endothelial dysfunction. The pharmacologic role of spironolactone in ameliorating endothelial dysfunction may involve the reversal of this pathway.

### Study limitations

Our study has several limitations. First, instead of screening all NOX subtypes, we examined only the interaction between mitochondrial ROS and NOX2. The main reason is that NOX2 is the prototype of NOX [29] and has been proposed to influence endothelial dysfunction in humans. [30] Second, increased cytosolic oxidative stress may not be generated only from mitochondria and NOX, we cannot exclude the possibility that intracellular oxidative stress originated from other pathways such as xanthine oxidase, lipoxygenase, cyclooxygenase, and uncoupled eNOS. However, a previous study suggests that spironolactone enhances eNOS protein expression through inhibiting NOX, not xanthine oxidase or uncoupled eNOS [31]; in addition, the interaction between mitochondrial ROS and NOX is a well known model for RIRR. [28] Therefore, we propose that spironolactone may attenuate the AGEs/RAGE-induced endothelial dysfunction through inhibiting cytosolic ROS generation from mitochondria and NOX. Third, uremic toxins are not limited to AGEs; thus, we cannot exclude the possibility that spironolactone can ameliorate endothelial dysfunction induced by uremic toxins other than AGEs. Fourth, compared with Group 2, we failed to demonstrate significantly lower plasma creatinine level in Group 4. This implied that spironolactone failed to improve renal function in 5/6 radial nephrectomy renal failure model. However, due to limited numbers with inadequate statistical power, the interpretation of the study result should be cautious. In addition, numerically higher BUN/Creatinine ratio after the treatment in Group 4 was noted. This indicated that the renal protection effect of spironolactone may be further confounded by its diuretic effect. [32, 33] Fifth, we only provide evidence that spironolactone could improve endothelial dysfunction in a CKD rat model. We strongly recommend that the beneficial effects of spironolactone on cardiovascular outcomes should be weighed against the potential risk of electrolyte imbalance, particularly in the setting of chronic renal failure.

## Conclusions

**Fig. 9:**
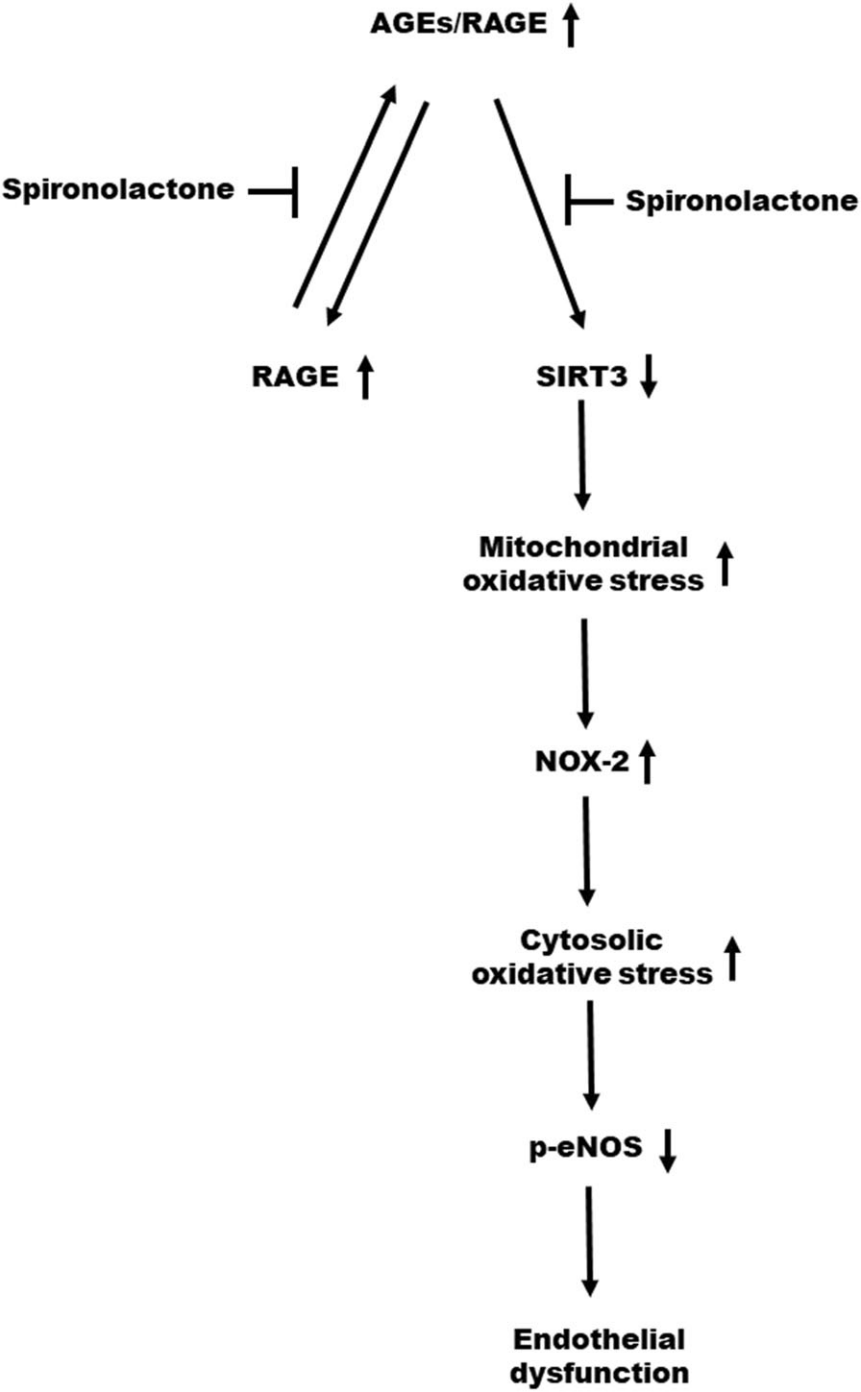
Proposed mechanism through which spironolactone ameliorates endothelial dysfunction in the setting of chronic kidney disease; the mechanism involves inhibiting the activation of advanced glycation end products (AGE)/receptor for AGEs (RAGEs) axis. Spironolactone can inhibit the positive feedback loop of AGEs-induced RAGEs formation. Spironolactone can ameliorate endothelial dysfunction through a reversal of the downregulation of sirtuin-3 (SIRT3). SIRT3 can regulate mitochondrial oxidative stress. Reduced SIRT3 increases mitochondrial oxidative stress, which further increases the nicotinamide adenine dinucleotide phosphate oxidase (NOX-2) production, forming reactive oxygen species (ROS)-induced ROS release (RIRR).The overall increased oxidative stress ultimately results in endothelial dysfunction

Spironolactone could ameliorate endothelial dysfunction in a 5/6 nephrectomy renal failure model. The possible mechanism involves the inhibition of the AGEs/RAGE axis, upregulation of SIRT3, and attenuation of both NOX-2 and its associated intracellular oxidative stress. (Fig. 9) Our study implied that spironolactone, a mineralocorticoid receptor antagonist, may be vasoprotective in the setting of renal failure.

## Additional files

Additional file 1: Immunohistochemical stain of thoracic aorta of Sprague-Dawley rats was illustrated above. (A) The amount of receptor for advanced glycation end products (RAGE) in thoracic aorta tissue is higher in the chronic kidney disease (CKD) group compared with that in the control, the CKD + ALT-711 (advanced glycation end products [AGEs] breaker), and the CKD + spironolactone (SPL) groups. (B) The amount of endothelial nitric oxide synthase (eNOS) in the thoracic aorta tissue is higher in the control group than that in the CKD, the CKD + ALT-711, and CKD + SPL groups; while the amounts of eNOS are comparable between the three groups. (JPG 1371 kb)

Additional file 2: Comparisons of cell viability in vitro between different concentrations of (A) advanced glycation end products (AGE) and (B) Bovine serum albumin (BSA) and (C) Spironolactone (SPL). * any groups vs. vehicle, *p* < 0.05. (JPG 504 kb)

## Data Availability

The datasets analysed during the current study are available from the corresponding author on reasonable request (d19863@mail.cmuh.org.tw).

## Abbreviations

Ach: Acetylcholine
AGEs: Advanced glycation end products
ANOVA: Analysis of variance
BSA: Bovine-serum albumin
BUN: Blood urea nitrogen
CKD: Chronic kidney disease
Cr: Creatinine
DAPI: 4′, 6′-diamidino-2-phenylindole
DCFH-DA: 2′,7′-Dichlorodihydrofluorescein diacetate
DMSO: Dimethyl sulfoxide
ECL: enhanced chemiluminescence detection
eNOS: endothelial nitric oxide synthase
HAECs: Human aortic endothelial cells
HRP: horseradish peroxidase
L-NMMA: N(omega)-monomethyl-L-arginine acetate
MTT: 3-(4,5-Dimethylthiazol-2-yl)-2,5-diphenyltetrazolium bromide
NOX: nicotinamide adenine dinucleotide phosphate oxidase
PBS: phosphate-buffered saline
PVDF: Polyvinylidene difluoride
RAGE: Receptor for advanced glycation end products
RIRR: ROS-induced ROS release
ROS: reactive oxygen species
SDS-PAGE: sodium dodecyl sulphate-polyacrylamide gel electrophoresis
SIRT3: sirtuin-3
SNP: Sodium nitroprusside
