# Supplementary figures and images for: Spironolactone ameliorates endothelial dysfunction through inhibition of the AGE/RAGE axis in a chronic renal failure rat model
Source: BMC Nephrol. 2019 Sep 6;20:351. doi: 10.1186/s12882-019-1534-4 (PMC6729054; doi:10.1186/s12882-019-1534-4)

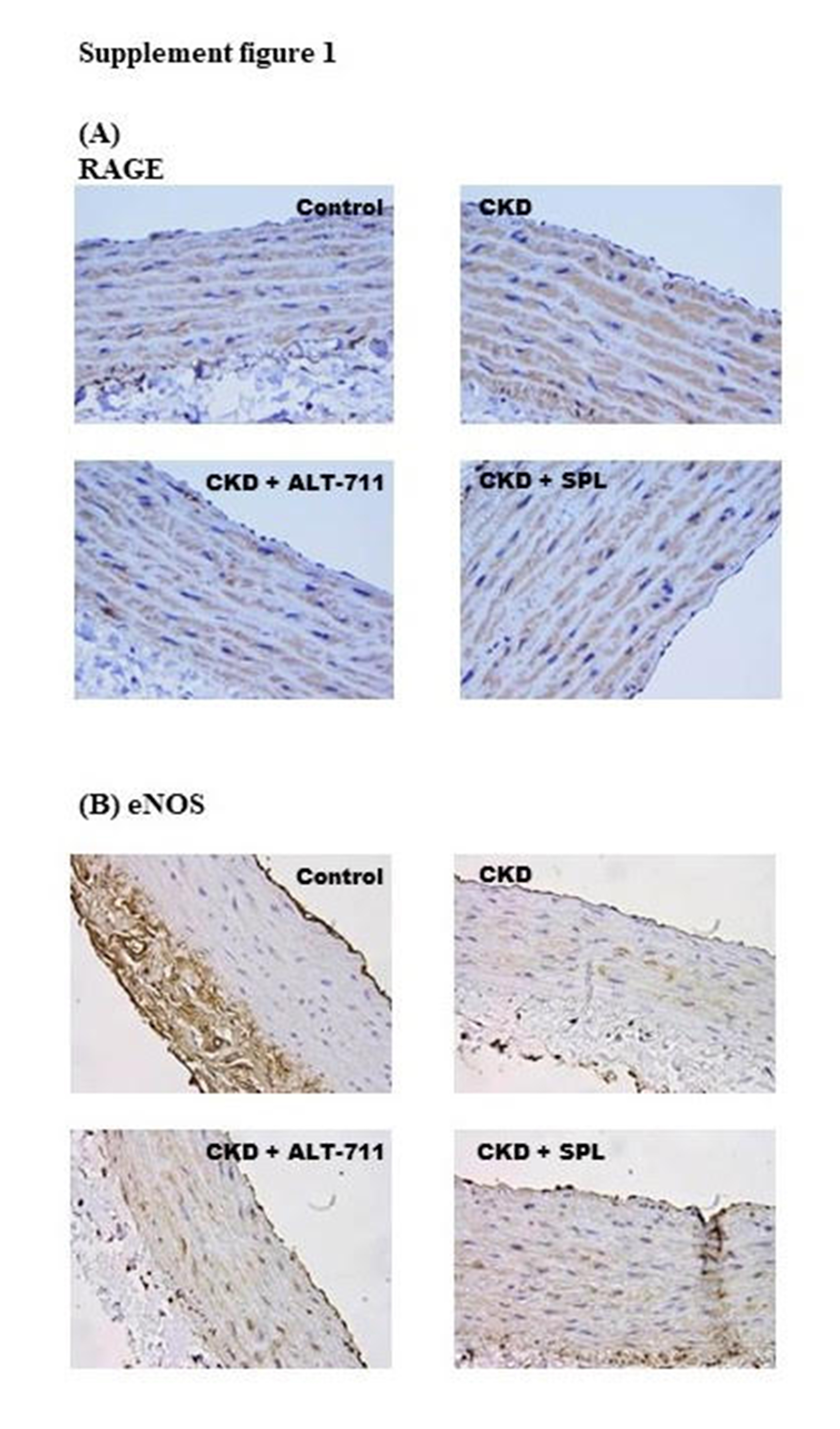

Supplement: Supplementary file 1 — Immunohistochemical stain of thoracic aorta of Sprague-Dawley rats was illustrated above. (A) The amount of receptor for advanced glycation end products (RAGE) in thoracic aorta tissue is higher in the chronic kidney disease (CKD) group compared with that in the control, the CKD + ALT-711 (advanced glycation end products [AGEs] breaker), and the CKD + spironolactone (SPL) groups. (B) The amount of endothelial nitric oxide synthase (eNOS) in the thoracic aorta tissue is higher in the control group than that in the CKD, the CKD + ALT-711, and CKD + SPL groups; while the amounts of eNOS are comparable between the three groups. (JPG 1371 kb) [file 12882_2019_1534_MOESM1_ESM.jpg]

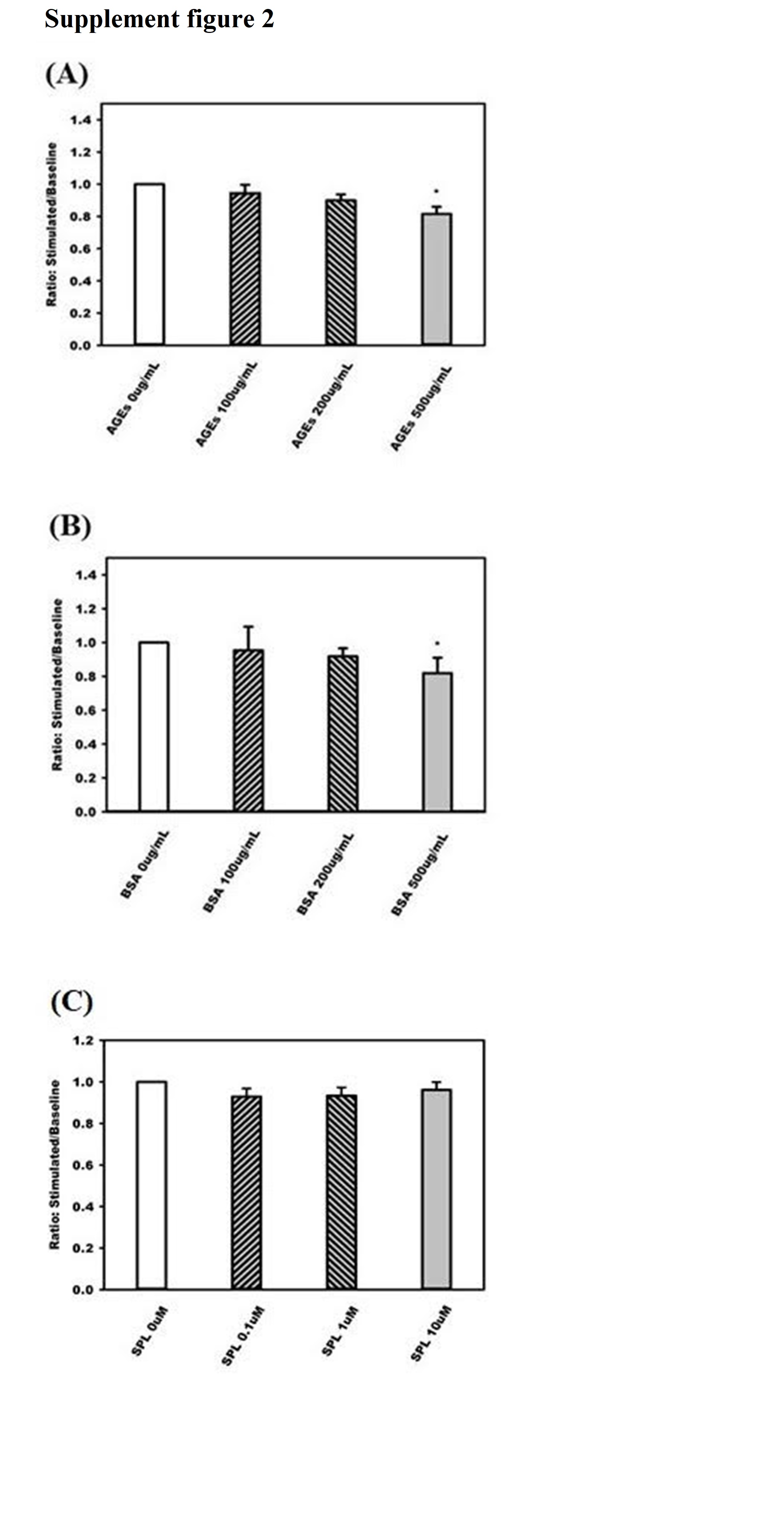

Supplement: Supplementary file 2 — Comparisons of cell viability in vitro between different concentrations of (A) advanced glycation end products (AGE) and (B) Bovine serum albumin (BSA) and (C) Spironolactone (SPL). * any groups vs. vehicle, p < 0.05. (JPG 504 kb) [file 12882_2019_1534_MOESM2_ESM.jpg]
